# Multifunctional Polymer-Metal Lattice Composites via Hybrid Additive Manufacturing Technology

**DOI:** 10.3390/mi14122191

**Published:** 2023-11-30

**Authors:** Liu He, Peiren Wang, Lizhe Wang, Min Chen, Haiyun Liu, Ji Li

**Affiliations:** 1 Key Laboratory of MEMS of the Ministry of Education, Southeast University, Nanjing 210096, Chinawang_peiren@seu.edu.cn (P.W.); 2 School of Advanced Technology, Xi’an Jiaotong-Liverpool University, Suzhou 215123, China; 3 College of Computer and Information, Hohai University, Nanjing 211100, China

**Keywords:** additive manufacturing, electroless plating, electroplating, lattice composite, multifunctional

## Abstract

With increasing interest in the rapid development of lattice structures, hybrid additive manufacturing (HAM) technology has become a competent alternative to traditional solutions such as water jet cutting and investment casting. Herein, a HAM technology that combines vat photopolymerization (VPP) and electroless/electroplating processes is developed for the fabrication of multifunctional polymer-metal lattice composites. A VPP 3D printing process is used to deliver complex lattice frameworks, and afterward, electroless plating is employed to deposit a thin layer of nickel-phosphorus (Ni-P) conductive seed layer. With the subsequent electroplating process, the thickness of the copper layer can reach 40 μm within 1 h and the resistivity is around $1.9\times 10^{-8}\;\Omega \cdot m$, which is quite close to pure copper ($1.7\;\times 10^{-8}\;\Omega \cdot m$). The thick metal shell can largely enhance the mechanical performance of lattice structures, including structural strength, ductility, and stiffness, and meanwhile provide current supply capability for electrical applications. With this technology, the frame arms of unmanned aerial vehicles (UAV) are developed to demonstrate the application potential of this HAM technology for fabricating multifunctional polymer-metal lattice composites.

## 1. Introduction

The lattice structure is made up of a series of spatially periodic units [1]. The lattice structure provides the flexibility to achieve different physical properties such as a high stiffness–weight ratio [2], negative Poisson’s ratio [3], ultra-low density [4], and low thermal expansion coefficient [5], and is therefore used in a wide variety of applications [6,7,8,9,10]. Conventional fabrication techniques (e.g., water jet cutting [11], investment casting [12], and wire-woven methods [13]) have been used to create lattice structures. However, these methods rely on complex equipment and require secondary assembly.

Additive manufacturing (AM) technology creates 3D objects layer by layer based on computer-aided design (CAD) files, which offers high freedom of design and appropriate processing methods for 3D printing products. AM has become a popular method for manufacturing lattice structures that can reduce generation costs and shorten processing time. For instance, Traugutt et al. [14] used Digital Light Processing (DLP) 3D printing to fabricate lattice devices that have 12 times greater rate-dependence and up to 27 times greater strain energy dissipation compared to commercial photopolymers. Wang et al. [15] fabricated small-scale microlattices via two-photon lithography (TPP) and large-scale microlattices by projection microstereolithography (PµSL), respectively. Unfortunately, polymer-based 3D-printed lattice structures normally have low mechanical strength, while their robust metal counterparts are more expensive to build. Moreover, most applications have only utilized the mechanical properties of lattice structures, and have not equipped them with other functionalities, which limits the application potential of lattice structures.

Hybrid additive manufacturing (HAM) technology is a significant improvement in AM technology that integrates complementary processes within an AM procedure [16]. With multiple processes, HAM can create lattice structures with different types of materials which enable the addition of extra functionalities.

Electroplating (EP) and electroless plating (/ELP) are also preferable complementary techniques in the HAM area, which can deposit functional metallic coatings on a 3D substrate for different purposes. For example, Zheng et al. [17] used large-area projection microstereolithography (LAPμSL) and electroless nickel deposition to fabricate nickel-phosphorus microlattices with high tensile (~20%) and compressive elastic deformation (>50%). Song et al. [18] fabricated nickel-coated polymer meso-lattice composites with fused deposition modeling (FDM) technology and electroless plating, which made the average modulus and strength increase by 68.3% and 34.9%, respectively, compared to the polymer lattice. Hensleigh et al. [19] employed SLA technology to print a multi-material structure with positive, negative, and neutral resins and soaked it in either a positive or negative catalyst solution to define the ELP deposition area for the fabrication of 3D electronics. Tang, Z et al. [20] combined AM and thermal growth processes to prepare elastomeric composite dots with microstructures that have excellent impact energy dissipation capabilities and thermal insulation properties. Our group proposed a series of HAM technology experiments based on laser activated electroless plating for the fabrication of 3D conformal/multilayer electronics, sensors, antennas, etc. [21,22,23,24,25].

**Figure 1:**
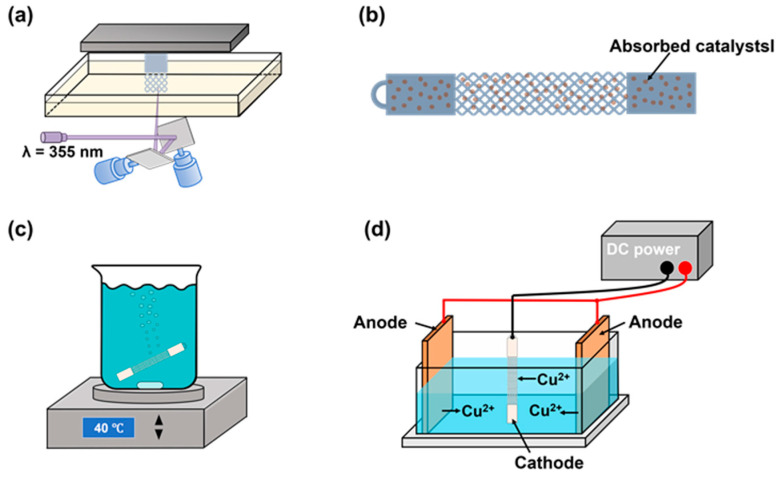
The fabrication process of the proposed HAM technology. (**a**) Stereolithography 3D printing. (**b**) Surface treatment and activation. (**c**) Electroless nickel plating. (**d**) Electroplating copper.

In this study, we further exploit the advantages of electro/electroless plating-based HAM for the development of multifunctional polymer-metal lattice composites. As shown in Figure 1, the lattice frameworks are via a high-precision VPP 3D printing process. After a series of pretreatment steps, nickel-phosphorus (Ni-P) film is electroless plated on the surface of the photopolymer lattice framework. With the conductive Ni-P film, electroplating can be conducted to thicken the metallic shell, which dramatically enhances the mechanical performance of the lattice structure. The thick metal shell also provides much lower resistance, and thereby could be used for electrical interconnection instead of wires. Moreover, the metal coating mitigates the aging of photopolymer materials caused by moisture absorption and ultraviolet (UV) irradiation in sunlight. To fully demonstrate the application potential of the multifunctional polymer-metal lattice composites, the frame arms of an unmanned aerial vehicle (UAV) with mechanical enhancement and current supply capability were developed and tested in this study.

## 2. Methods

### 2.1. Process Chain of the HAM

#### 2.1.1. VPP 3D Printing

The experimental samples in this paper were designed via Cero 8.0 software (PTC, Boston, MA, USA) and fabricated with industrial-grade VPP 3D printers (Lite 600HD, UnionTech, Shanghai, China). The resin we used was a commercial photopolymer (SH8801, UnionTech, Shanghai, China). To guarantee high precision of fabrication, the layer thickness of the key printing parameter for VPP 3D printing was set to a minimum value of 50 μm. After printing, the samples were cleaned with organic solvents and deionized water and then subjected to a secondary curing process with 365 nm UV light.

#### 2.1.2. Electroless Plating

The electroless nickel plating process involves the following main steps.

(1) Etching: The samples were first immersed in a strong oxidizing corrosive solution (60 g/L potassium permanganate (KMnO_4_, >99.5%) and 30 g/L sodium hydroxide (NaOH, >99.5%)) at 50 °C for 1 min to improve the surface roughness. The polymer samples were then placed in a reducing agent bath (75 mL/L hydrochloric acid (HCl, 36.0–38.0%) and 60 mL/L hydrazine hydrate (N_2_H_4_·H_2_O, >85.0%)) for about 3 h to remove residual strong oxidants.
(2) Sensitization-activation: The lattice frameworks were sensitized and activated in a mixture of 60 g/L sodium chloride (NaCl, > 99.5%), 230 mL/L HCl, and 10 mL/L concentrated palladium–tin colloid (Act PP-950) for 10 min. In this step, Pd^2+^ was reduced to Pd^0^ metallic catalytic particles in the presence of Sn^2+^.
(3) Acceleration: The samples were soaked in a dilute acid solution (10 vol% HCl) at 40 °C for 5 min to wash away the excess colloid and expose the Pd^0^ particles.
(4) Plating: The alkaline nickel-plating solution consists of 20 g/L of nickel sulfate heptahydrate (NiSO_4_·7H_2_O, >99.5%), 30 g/L of sodium hypophosphite monohydrate (NaH_2_PO_2_·H_2_O, >99.5%), and 10 g/L of sodium citrate (Na_3_C_6_H_5_O_7_, >99.5%). The plating process was carried out at 38 °C for 15 min, and the pH of the solution was adjusted to about 8.5 by using ammonia (NH_3_·H_2_O, 25–28%).

The samples were rinsed with deionized water after each of the above steps, and the steps not otherwise specified were carried out at room temperature.

#### 2.1.3. Electroplating

Before electroplating, the Ni-P coated lattices were activated in 5 vol% sulfuric acid (H_2_SO_4_, 95.0–98.0%) solution for 30 s to remove the oxide film on the surface. The electrodeposition of copper was performed in a Haring cell (working volume 1500 mL) with a bubble agitation system and copper anodes (phosphorus content: 0.03–0.05%) on both sides. The Ni-P coated lattice was connected to the cathode and placed in the middle of the Haring cell. The EP copper bath contained 200 g/L copper sulfate pentahydrate (CuSO_4_·5H_2_O, >99.0%), 30 mL/L H_2_SO_4_, 60–100 ppm chlorine ions (Cl^−^), and organic additives such as brightener and leveler). A direct current (DC) was applied and the current density (the applied electric current divided by the total conducting area of the sample immersed in the EP bath) was set to a low value of 2 A/dm^2^. Although low current density caused slow plating speed, the induced Cu grains were fine and uniform, which provided a dense and smooth copper layer [26]. Finally, the samples were dipped in an organic passivation solution (C-901 Copper Antioxidant, Rongxin Co., Ltd., Dongguan, China) for 5 min at room temperature to prevent oxidation of the copper layer. To avoid a potential electrical hazard, the organic passivation layer coated on the copper surface could act as electrical isolation layer. The plating process was performed for 0.5, 1.0, 1.5, and 2.0 h at 25 °C, respectively. The thickness of the coating was measured to identify the plating speed.

### 2.2. Sample Design and Simulation

#### 2.2.1. Design of Test Specimens

To investigate the mechanical enhancement of metal coating, tests samples for mechanical testing, including tensile, compression, and bending, were designed referring to ASTM D638-14 [27], ASTM D695-15 [28], and ASTM D790-17 [29] standards, respectively.

**Figure 2:**
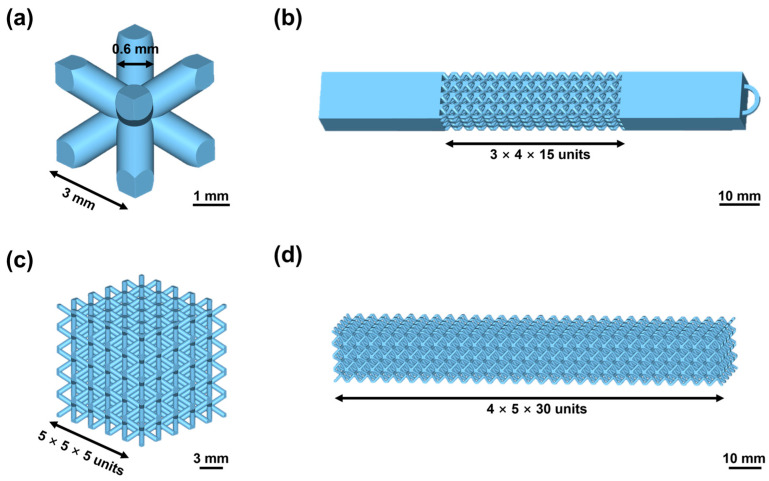
Diagram of the lattice structures. (**a**) The structure of the lattice unit cell. The structural design drawings of (**b**) tensile, (**c**) compression, and (**d**) bending samples.

The dimension of each lattice cell was designed as 3 mm × 3 mm × 3 mm (Figure 2a) and the strut diameter was set to 0.6 mm. For tensile samples, a BCC lattice structure with clamping parts at both sides was designed, as shown in Figure 2b. The number of lattice cells in length, width, and height direction was 4, 3, and 15, respectively. The dimensions of both clamping parts were 30 mm × 9 mm × 12 mm. The small ring at one end was used for the fixture during the plating process. For compression and bending samples, a 3 mm × 3 mm × 3 mm BCC lattice cell was also used, and the cell numbers in three dimensions were 5 × 5 × 5 (Figure 2c) and 4 × 5 × 30 (Figure 2d), respectively.

#### 2.2.2. Numerical Simulation

Simulating structural components made of micro/mesoscale lattices presents significant challenges due to the considerable modeling and computational expenses involved. Drawing from the multiscale principle, the minimal repeatable structure is termed the representative volume element (RVE) [30]. This RVE should be sufficiently small to remain unaffected by parameters of a larger scale, yet adequately large to satisfy the research geometry parametrization criteria [31,32]. In this framework, homogenization theory efficiently evaluates volume-weighted stress and strain values. It posits that homogenized properties parallel those of a homogeneous material, closely mirroring the response of its heterogeneous periodic counterpart. For RVEs containing voids, many studies use a single unit cell as the RVE. Here, the impact of RVE size is taken into consideration and the unit cell number within a single RVE is determined as 3 × 3 × 3 [33].

With the homogenization method, it is essential to apply periodic boundary conditions (PBCs) appropriately to the RVE during load-induced deformation. In the present study, the PBCs with uniform strain conditions are implemented for coated BCC lattice RVEs. Therefore, the general boundary conditions are applied with:

(1) $$\begin{array}{c} u_{i}+u_{i}^{\prime }=U_{i}^{0}\\ u_{i}=0\\ u_{i}^{\prime }=U_{i}^{0} \end{array}$$

where $u_{i}$ and $u_{i}^{\prime }$ represent the periodic surfaces of the lattice RVE and $U_{i}^{0}$ describes the global displacement of the RVE. The applied PBC, using the uniform strain approach, not only constrains the RVE’s deformation but also ensures stress and geometrical compatibility within the RVEs through stress and strain constraints.

For numerical modeling of the coating film, Shell 281 elements with the consideration of membrane and bending stiffness behaviors are utilized. This approach drastically reduces the number of elements post-discretization compared to solid elements, while maintaining high precision [32].

### 2.3. Characterization

#### 2.3.1. Mechanical Properties Test

The tensile, compression, and bending tests were performed using a universal test machine (UTM 2503, Suns Technology Stock Co., Ltd., Shenzhen, China). For each test group, 5 samples were prepared to obtain the force–displacement curves. The curves were transformed into stress–strain curves according to the equation below

(2) $$\sigma_{s}=\frac{F}{A}$$

(3) $$\varepsilon =\frac{\Delta l}{l}$$

where F is the uniaxial reaction force caused by the input loading, Δl is the uniaxial external displacement, and l and A represent the dimension and cross-sectional area of the lattice structure.

Stiffness is the ability of a material or structure to resist elastic deformation when subjected to a force. Here, the elastic stiffness of the lattice structures concerning tension, compression, and bending, denoted by $k_{t}$, $k_{c}$, and $k_{b}$ correspondingly, is described in the following normalized way:

(4) $$k_{t,c,b}=\frac{F_{elastic}}{\delta }$$

where $F_{elastic}$ represents the maximum reaction force applied to the lattices during the elastic deformation phase and δ stands for the corresponding generated elastic displacement.

The structure ductility indicates the ability of a material to deform plastically before rupture occurs due to force. Based on the transform from the force–displacement curve to engineering stress–strain relationship with Equations (2) and (3), the energy absorption capacity *W* can be expressed as:

(5) $$W=\int_{0}^{\varepsilon }\sigma (\varepsilon )\;d\varepsilon$$

Firstly, the mechanical strength of the post-cured samples was measured according to the ASTM D638-14 standard, and a Type IV test specimen was prepared in this study. The post-curing process was performed for 0, 5, 15, 30, 45, 60, 90, and 120 min, respectively. The speed of testing was set to 1 mm/min.

Secondly, to investigate the effect of the nickel ELP process on the mechanical property, 5 lattice samples (Figure 2b) were prepared for each ELP step such as etching, sensitization-activation, acceleration, and plating. Original lattice samples were also tested as a benchmark. The tensile speed of the test machine was set to 1 mm/min.

Thirdly, to systematically explore the influence of different shell thicknesses on the mechanical strength of metal-polymer composite lattice, lattice structures (Figure 2b) with 10, 30, 50, and 80 μm thick copper coating were prepared, and 5 samples of each thickness were first tensile tested to obtain their force–displacement curves. With the comprehensive assessment of above-mentioned factors, an optimal thickness could be acquired. This optimal coating thickness was also adopted for compression and bending samples.

#### 2.3.2. Electrical Properties of Metallic Coatings

The electrical conductivity of metallic materials is critical for their electrical applications, which can be calculated according to Pouillet’s law as follows:

(6) $$\sigma_{c}=\frac{1}{\rho }=\frac{R\cdot A}{L}=\frac{R\cdot w\cdot d}{L}$$

where *σ_c_* is the conductivity of the conductor, *ρ* is the resistance of the conductor, *R* (Ω), *A* (cm^2^), *L* (cm), *w* (cm), and *d* (cm) are the resistance, cross-section area, length, width, and thickness of the conductor, respectively. In this test, 2 mm × 15 mm Ni-P and copper layers were fabricated for the measurement of conductivity. The *R* was measured via a four-point probe method, the *d* was measured via an electrolytic metal thickness gauge, and the *L* and *w* were obtained via an optical microscope, respectively.

#### 2.3.3. Material Characterizations

The tensile, compression, and bending tests were performed using a universal test machine (UTM 2503, Suns Technology Stock Co., Ltd., Shenzhen, China).

The scanning electron microscope (SEM) imaging and energy-dispersive spectroscopy (EDS) mapping were conducted using a field-emission scanning electron microscope (FE-SEM) (FE-SEM, Ultra Plus, Carl Zeiss, Jena, Germany) with integrated EDS (X-Max 20, Oxford Instrument, Oxford, UK) for indicating the microstructure and element distribution. To investigate the cross-section, lattice structures with nickel or copper layers were first cut with a razor blade and then mounted by casting with epoxy polymer at room temperature (cold mounting). After the cold mounting resin solidified, the mounted specimens were then progressively ground and polished to form a mirror-like surface. Subsequently, a thin layer of gold was sputtered on its surface to avoid charge accumulation during the SEM study.

The X-ray photoelectron spectroscopy (XPS) analysis of the valence state changes of the Pd element was carried out using an X-ray photoelectron spectrophotometer (PHI 5000 VersaProbe, ULVAC-PHI Inc., Kayasaki, Japan).

The surface topography of nickel-coated lattice structures and copper-coated lattice structures and surface roughness were characterized via a chromatic confocal profilometer (JR 25, Nanovea, Irvine, CA, USA). The step size selected here was 5 µm in the X-direction and the Y-direction.

An electrolytic metal thickness gauge based on the Faraday electrolysis principle (DJH-G, Research Institute of Materials Protection, Wuhan, China) was used to measure the thickness of the copper layer. The samples were fixed on the electrolytic metal thickness gauge and the S4 (JH-G, Research Institute of Materials Protection, Wuhan, China) electrolyte was poured into the electrolytic cup. The thickness and the potential change were monitored in real time on a computer. A total of 4 points on each side were selected to measure the thickness of each sample average.

## 3. Results and Discussion

### 3.1. Manufacturing Results of the HAM

#### 3.1.1. ELP Process

**Figure 3:**
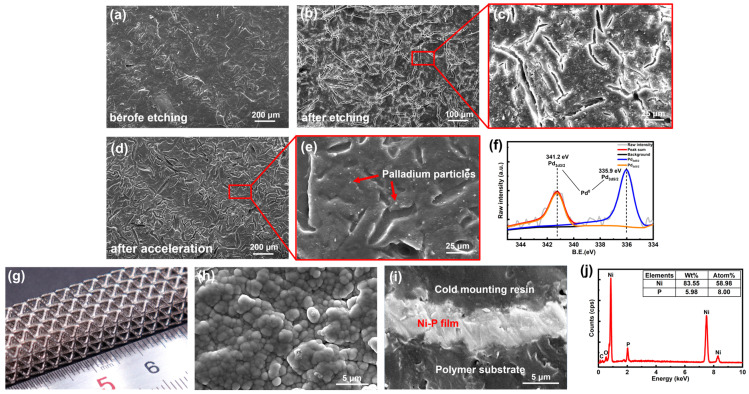
The characterization of the ELP process. The SEM images of (**a**) before etching, (**b**,**c**) after etching, and (**d**,**e**) after the acceleration step. (**f**) The XPS result of the Pd element of (**d**). (**g**) The photo of the nickel-coated lattice structure. (**h**,**i**) The SEM image of the nickel layer surface and the cross-section after 150 min ELP. (**j**) The EDS result of the nickel layer.

In order to increase the roughness and hydrophilicity of the polymer surface, a strong oxidative KMnO_4_ solution was used in the etching step. By comparing the SEM images before and after the etching step (Figure 3a–c), it can be seen that the polymer surface was left with uniform cracks. During the acceleration step, the following reactions occurred to produce metallic palladium catalytic particles.

(7) $${\mathrm{Sn}}^{2+}+{\mathrm{Pd}}^{2+}\to {\mathrm{Sn}}^{4+}{+\mathrm{Pd}}^{0}$$

The metallic palladium particles were uniformly distributed on the polymer surface as shown in Figure 3d,e. Additionally, the XPS results identified the valence states of Pd elements (Figure 3f). The spectra were corrected by the C1s peak at 284.8 eV. Pd 3d peaks were composed of Pd 3d_5/2_ (335.9 eV) and Pd 3d_3/2_ (341.2 eV) which corresponded to Pd^0^ [34]. Nickel ions were reduced to nickel by the catalytic action of metallic palladium particles, followed by an autocatalytic reaction for continuous nickel plating. At the same time, secondary reactions occurred between hypophosphates and hydrogen atoms to produce elemental phosphorus.

(8) $${\mathrm{Ni}}^{2+}{+H}_{2}{\mathrm{PO}}_{2}{}^{-}+3{\mathrm{OH}}^{-}\overset{\mathrm{Pd}/\mathrm{Ni}}{\to }\mathrm{Ni}+{\mathrm{HPO}}_{3}{}^{2-}+2H_{2}O$$

(9) $$H_{2}{\mathrm{PO}}_{2}{}^{-}\overset{\mathrm{Pd}/\mathrm{Ni}}{\to }{\mathrm{PO}}_{2}{}^{-}+2H$$

(10) $$H_{2}{\mathrm{PO}}_{2}{}^{-}+H\overset{\mathrm{Pd}/\mathrm{Ni}}{\to }P{+\mathrm{OH}}^{-}+H_{2}O$$

After the reactions, a bright and dense Ni-P layer was deposited on the surface of the polymer (Figure 3g). The microstructure of the surface and cross-section of Ni-P film was investigated via SEM imaging. As shown in Figure 3h, the Ni-P layer was dense with no cracks. After 150 min ELP, the thickness of the Ni-P layer reached around 3.7 μm (Figure 3i), and the resistivity of the nickel layer was approximately $8.2\times 10^{-7}\Omega \cdot m$. EDS investigation illustrated that the concentration of phosphorus and nickel in the alloy film was 5.98 wt% and 83.55 wt%, respectively (Figure 3j).

#### 3.1.2. EP Process

Electroplating (EP) is a method of applying a layer of metal to the surface of a conductive object using the principle of electrolysis [35]. Compared with its ELP counterpart, EP can rapidly deposit a thick metallic layer on the conductive lattice frame. In this way, the mechanical strength of the polymer-metal lattice composite structures could be largely enhanced, and, meanwhile, the manufacturing cost was much less than pure metal lattices. Moreover, the thick metal shell also provides higher conductivity and thereby could be utilized for electrical interconnection instead of replacing electric wires. In this work, the thick copper coating was adopted for both mechanical enhancement and current supply for the application of UAVs.

**Figure 4:**
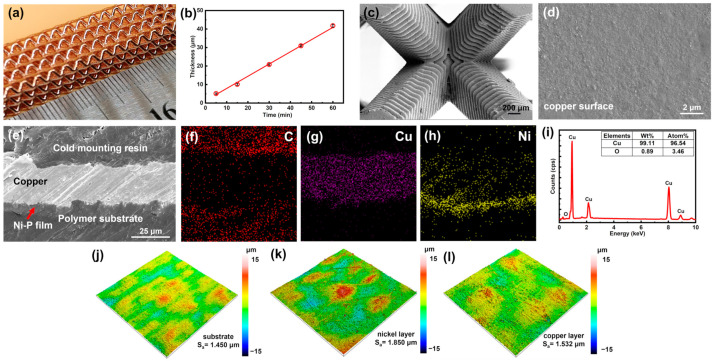
The characterization of the copper layer. (**a**) Photo of the copper-coated lattice structure. (**b**) The plating speed of the electroplating copper. SEM images of (**c**) the morphology of the lattice structure, (**d**) the copper layer surface, and (**e**) the cross-section of the copper layer. Elemental mapping of (**f**) C, (**g**) Cu, and (**h**) Ni in the cross-section. (**i**) EDS result of the copper layer. (**j**–**l**) The surface morphology and roughness of the printed photopolymer, nickel layer, and copper layer.

The electroplated copper layer had a pinkish-orange color with a metallic luster (Figure 4a). The thickness of the copper layer increased linearly with plating time during the electroplating process, which was attributed to the fact that the concentration of copper ions (Cu^2+^) in the plating solution did not decrease. The following reactions continuously occurred at the cathode and anode during the plating process:

(11) $${\mathrm{Cu}}^{2+}+2e^{-}\to \mathrm{Cu}\;\left(\mathrm{cathode}\right)$$

(12) $$\mathrm{Cu}-2e^{-}\to {\mathrm{Cu}}^{2+}\;\left(\mathrm{anode}\right)$$

The anode copper lost electrons into Cu^2+^ entering the solution, and the Cu^2+^ near the cathode gained electrons into Cu deposited on the surface of the sample. Therefore, the concentration of Cu^2+^ in the solution remained theoretically constant. In order to control the thickness of the copper plating layer, the plating speed was first obtained by measuring the thickness of copper coatings produced with different plating times (Figure 4b). The plating speed was calculated as 0.67 μm/min. SEM was applied to investigate the cross-section and the surface of the copper-coated lattice. A dense copper shell was observed on the lattice structure, and the step feature was due to the layer lines caused by the 3D printing process (Figure 4c,d). Compared with Ni-P film, the thickness of the copper layer was much higher, and reached about 25 μm for about 37 min of electroplating (Figure 4e). The EDS mapping clearly showed the interfaces between the copper layer, Ni-P film, the mounting photopolymer, and the photopolymer substrate (Figure 4f–h). There were a few carbon and nickel elements smeared on the copper area, which might have been caused by the grinding and polishing during the sample preparation. According to the EDS result (Figure 4i), the purity of the copper layer was 99.11 wt% which ensured its low electrical resistivity. The resistivity of the copper layer reached a minimum value $1.9\times 10^{-8}\;\Omega \cdot m$ after 1 h of plating, which was close to the pure copper ($1.7\;\times 10^{-8}\;\Omega \cdot m$) [36]. The surface morphologies of the 3D-printed polymer, the nickel layer, and the copper layer were investigated via a chromatic confocal profilometer. A slight increase in average surface roughness (Figure 4j,k) after nickel plating could be detected, which was attributed to the strong corrosive effect of KMnO_4_ during the etching process. The deposition of the copper layer filled in the potholes on the surface, and this result indicated that the surface roughness of the copper layer (S_a_ = 1.532 µm) could be reduced via long-term (1 h) electroplating deposition (Figure 4l).

### 3.2. Effects of Post-Curing and Electroless Plating Steps on the Mechanical Strength of Lattice Structure

**Figure 5:**
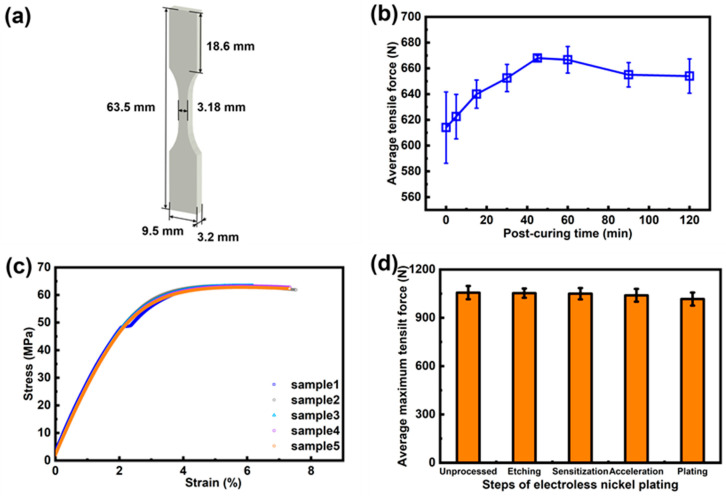
The characterization in the pre-treatment process. (**a**) The dimension chart of the Type IV specimen. (**b**) The mechanical properties of the printed structure during the different post-curing times. (**c**) Stress–strain curves at the post-curing time of 45 min. (**d**) The tensile strength of lattice samples after each ELP process step.

VPP 3D printing is based on the principle of photopolymerization to print 3D parts. After the printing process, the photopolymer is usually not fully cross-linked and thereby cannot provide its best mechanical performance. Moreover, with the further polymerization caused by UV light, the mechanical strength could be varied, which made it difficult to accurately evaluate the mechanical enhancement due to the ELP/EP process. Therefore, we employed a post-curing process to make the lattice samples achieve their highest mechanical strength,

As shown in Figure 5b, the original lattice samples (Figure 5a) had large deviations in tensile strength, which indicated that the degree of curing varied largely among the samples. With the further cross-link of photopolymers during the post-curing step, the tensile strength gradually increased from 614 N to 668 N, and the consistency of tensile strength in different samples was dramatically improved. On the contrary, when the post-curing period was longer than 45 min, the tensile strength was reduced slightly. This was attributed to material aging caused by over-curing. Therefore, proper post-curing treatment could enhance the curing degree of photopolymer samples and thereby provide a much more stable basic mechanical strength for subsequent investigation. Finally, the optimum post-curing time was chosen to be 45 min for all prepared samples. The elastic modulus $E_{re\sin}$ of the post-cured photopolymer can be obtained from the slope of its stress–strain curve in the elastic deformation region (Figure 5c). With 45 min post-curing time, the average elastic modulus of the photopolymer was 2.654 ± 0.1458 GPa, which was used as an input parameter for simulation.

Lattice test samples were prepared as described in Section 2.2.1 to investigate the effects of ELP steps on the mechanical strength (Figure 2b). With the conducting of each ELP step, the average maximum tensile force decreased slightly from 1056 N to 1016 N by only 3.79% (Figure 5d). This exhibited that the metalization process would not dramatically degrade the mechanical strength of the lattice structure, even when a corrosive KMnO_4_ solution was used to etch the photopolymer surface.

### 3.3. Effects of Electroplating on the Mechanical Properties of Lattice Structures

**Figure 6:**
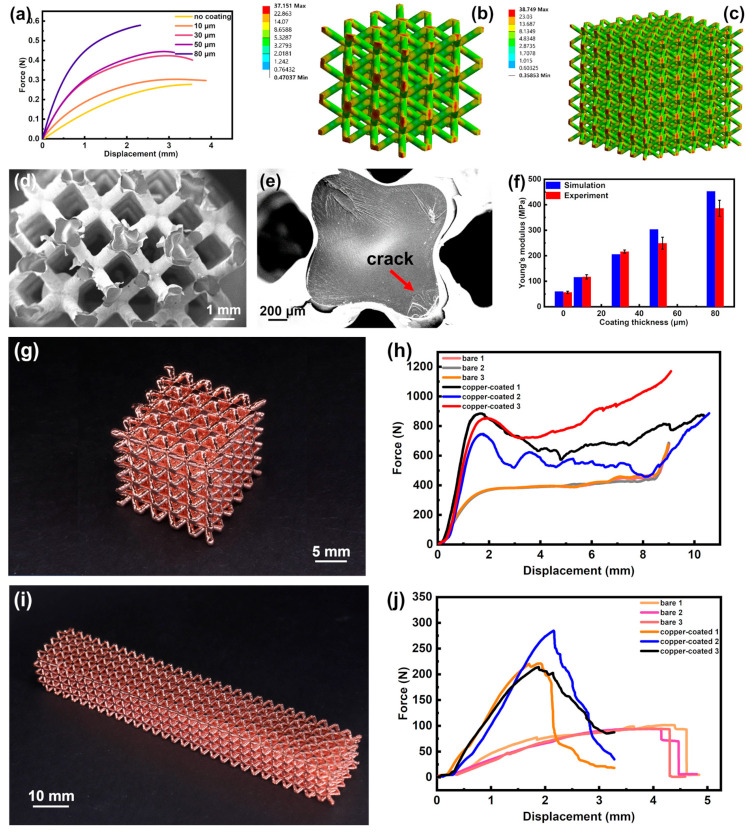
Testing and simulation of mechanical properties of lattice structures. (**a**) Force and displacement curves at different thicknesses. Simulation results of stress distribution of (**b**) 3 × 3 × 3 and (**c**) 6 × 6 × 6 lattice structure under 1 MPa stress. (**d**,**e**) The SEM image of lattice structure. (**f**) Simulation and experiment results of equivalent Young’s modulus. (**g**) The photo of compression sample and (**h**) its force–displacement curves. (**i**) The photo of bending sample and (**j**) its force–displacement curves.

The mechanical properties of the metal-coated lattice structure were assessed through tensile, compression, and bending tests, as depicted in Figure 6. To enhance the comparability between numerical simulations and experimental results, the mechanical properties, such as the elastic modulus (E), of both the resin and copper film, were determined using ANSYS Workbench 2020 R2. Specifically, the elastic modulus of the resin was obtained from stress–strain curves depicted in Figure 5c, derived from standard tensile tests detailed in our prior work [30]. Additionally, the elastic modulus of the copper was determined by analyzing variations in values within ANSYS Workbench. A comparison between the numerical results of coated lattice structures and experimental data was conducted for precise determination and validation. It is noteworthy that, due to the minimal deformation induced in the simulation, the lattice structure exhibited purely elastic behavior. Consequently, only elastic material properties were considered, and the detailed material information is presented in Table 1.

It is observed that the metal coating significantly improves the mechanical characteristics, including elastic stiffness (as shown in Figure 6a), energy absorption capacity (as evident in Figure 6h), and bending stiffness (as indicated in Figure 6j).

#### 3.3.1. Tensile Tests

Specifically, macroscopic experiments of copper-coated lattice structures were first performed based on BCC lattice structures with the framework of 3 × 4 × 15 to evaluate the significance of coating thickness (T) on mechanical characteristics, including maximum applied force ($F_{max}$) and equivalent Young’s modulus ($E_{lattice}$). As shown in Figure 6a, $F_{elastic}$ and $F_{max}$ both improved with the increased T, which revealed an enhancement of structural strength. Concerning structural stiffness, since the copper coating had a higher material density compared with the photopolymer matrix, the adhesion of the copper coating film impressively strengthened the structural stiffness, and it explained the monotonous enhancement of $k_{t}$ with increased T. In addition, for ductile properties, the lattice structures with T=10 μm and 30 μm coatings demonstrated the best ductility performances, rather than a pure photopolymer lattice matrix. Conversely, compared with the curve result of a pure photopolymer lattice matrix, the relatively thick coating negatively impacted the ductility of the whole structure.

Considering the balance between structural strength and ductility, a thickness of 30 μm was then chosen following compression and bending tests. For a further detailed explanation of the balanced performance that existed in the 30 μm-coated lattice structure, a numerical simulation and fracture surface analysis were performed.

Based on the multiscale numerical method utilized in our prior research [30], the von Mises stress contour of the lattice structure under the external displacement of 0.1 mm was described, as in Figure 6b. It showed excellent compatibility between the homogenization-based RVE results and the structural results at the global scale in Figure 6c. Moreover, the SEM images in Figure 6d,e described the exhibited striated fracture surfaces on the photopolymer matrix and cracks from the interface of adjacent layers of coated lattice structures with T=30 μm, which showed a good consistency with the maximum stress distribution in Figure 6a. Therefore, the rationality of the numerical method in assessing the lattice structure’s property was verified and could subsequently be employed to assess the elastic performance of coated lattice structures. As can be seen from Figure 6f, the results of $E_{lattice}$ obtained by simulation increased monotonously with larger T, and they were basically consistent with the results obtained via experiments.

#### 3.3.2. Compression Tests

In terms of compressive properties, a compression test was carried out on the 5 × 5 × 5 lattice structure (Figure 6g) with a speed of 5 mm/min, and the experimental results in Figure 6h illustrate that $k_{t}$ of copper-coated lattice structures was improved significantly compared with photopolymer lattice structures. Additionally, the W of copper-coated lattice structures was much larger than that of pure photopolymer-based lattice structures, which meant that the metal coating played a significantly positive impact on the energy absorption property of the lattice.

#### 3.3.3. Bending Tests

Moreover, to evaluate the bending characteristic, an experiment on coated lattice beams with dimensions of 4 × 5 × 30 (Figure 6i) was conducted on a three-point bending test. The lattice beam had a support span (L) of 80 mm, a width (b) of 15 mm, and a thickness (d) of 12 mm. The beams were loaded at a rate of 5 mm/min until failure. Figure 6j presents the force–displacement curves for both coated and uncoated lattice structures. From this, it was evident that the lattice structure with the coating film exhibited a greater $k_{b}$ and bending strength compared to its uncoated counterparts. Furthermore, when considering the $F_{max}$, the load-bearing capacity of the coated lattice structures surpassed that of the pure photopolymer-based lattices by more than double.

### 3.4. Demonstrator

**Figure 7:**
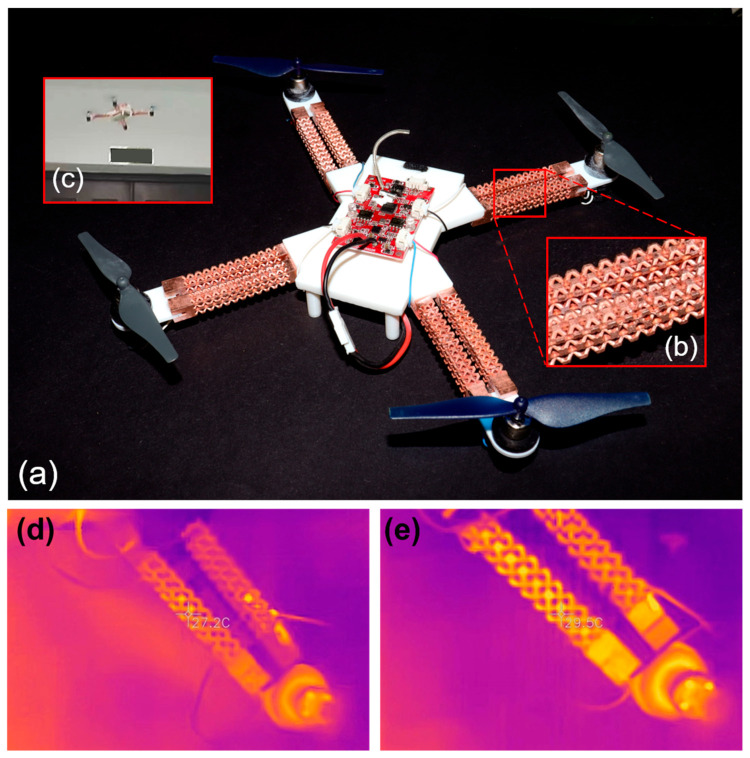
UAV demonstrator. (**a**) Full view of the UAV. (**b**) The details of the multifunctional lattice arms. (**c**) The photo of the flying UAV. Infrared images of the frame arms (**d**) before and (**e**) after the flight.

The frame arms of unmanned aerial vehicles (UAV) were successfully developed as an application demonstrator of multifunctional polymer-metal lattice composites (Figure 7a–c). There are four remarkable advantages in applying multifunctional polymer-metal lattice composites in UAVs. First, the metal-coated photopolymer lattice can largely decrease the structure weight and meanwhile provide the equivalent mechanical properties. Secondly, when suffering from any damage, the lattice structure possesses a discontinuous fracture mechanism to avoid the expansion of cracks. Thirdly, the thick metal layer restrains the aging of the photopolymer caused by humidity, UV radiation, chemical corrosion, etc. Lastly, the highly conductive copper layer could also be used for supplying current to the motors instead of the wires, which reduced the total weight and heat power loss. The electrical stability of the conductor was mainly affected by the carrying current, which might heat the copper conductors and thereby increase their resistance. An IR camera was used to measure the temperature before and after applying electrical current (the UAV was fixed on the table). The temperature increased from 27.2 °C to 29.5 °C (Figure 7d,e). Considering the air convention cooling during flying, the temperature change of the copper conductor could be ignored and thereby the resistance of the conductors is also unchanged.

## 4. Conclusions

In this work, we proposed a novel hybrid additive manufacturing method that integrates high-precision VPP 3D printing and EP/ELP metalization processes for the fabrication of multifunctional polymer-metal lattice composites. After VPP 3D printing, the photopolymer was etched for palladium catalyst attachment and then ELP of Ni-P was conducted to make a conductive seed layer for subsequent electroplating. A thick copper layer could then be rapidly (0.67 μm/min) EP deposited on the lattice structure, which significantly enhanced the mechanical properties. The electrical conductivity of copper could approach $1.9\times 10^{-8}\;\Omega \cdot m$ close to that of pure copper. Based on the proposed HAM technology, UAV frame arms with enhanced mechanical strength and current supply capability were successfully developed to verify the application potential of multifunctional polymer-metal lattice composites. In conclusion, our proposed approach provides a promising alternative to traditional fabrication methods for creating lattice structures and meanwhile endues the lattice structures with multiple extra functionalities.

## Figures and Tables

**Table 1:** The mechanical properties of resin base and copper film.

Resin		Copper	
Young’s Modulus E (GPa)	Poisson’s Ratio υ	Young’s Modulus E (GPa)	Poisson’s Ratio υ
2.62	0.3	105	0.34

## Data Availability

Data are contained within the article.
